# Adaptive Compression of Slowly Varying Images Transmitted over Wireless Sensor Networks

**DOI:** 10.3390/s100807170

**Published:** 2010-07-29

**Authors:** George Nikolakopoulos, Dionisis Kandris, Anthony Tzes

**Affiliations:** 1 Department of Electrical and Computer Engineering, University of Patras, Rio 26500, Greece; E-Mail: tzes@ece.upatras.gr (A.T.); 2 Department of Electronics, Technological Educational Institute of Athens, Athens 12210, Greece; E-Mail: dkandris@teiath.gr

**Keywords:** wireless multimedia sensor networks, control over networks, adaptive image compression, quad tree decomposition

## Abstract

In this article a scheme for image transmission over Wireless Sensor Networks (WSN) with an adaptive compression factor is introduced. The proposed control architecture affects the quality of the transmitted images according to: (a) the traffic load within the network and (b) the level of details contained in an image frame. Given an approximate transmission period, the adaptive compression mechanism applies Quad Tree Decomposition (QTD) with a varying decomposition compression factor based on a gradient adaptive approach. For the initialization of the proposed control scheme, the desired a priori maximum bound for the transmission time delay is being set, while a tradeoff among the quality of the decomposed image frame and the time needed for completing the transmission of the frame should be taken under consideration. Based on the proposed control mechanism, the quality of the slowly varying transmitted image frames is adaptively deviated based on the measured time delay in the transmission. The efficacy of the adaptive compression control scheme is validated through extended experimental results.

## 1. Introduction

The high disposability of sensors capable of capturing video/audio streams, stand still images and scalar sensor data enhanced the development of the so-called Wireless Multimedia Sensor Networks (WMSNs), which are networks of wirelessly interconnected sensor nodes able to retrieve and handle multimedia content. Their operation can be considered as the convergence between the classical wireless sensor networks (WSNs) and distributed multimedia acquisition devices (e.g., cameras, microphones, *etc.*). Nowadays, WMSNs gain an ever increasing share in scientific research in conjunction with the rapid developments and increasing miniaturization in the field of embedded systems [1,2].

Classical features of WSNs such as: (a) the easiness of the *ad hoc* deployment, (b) the ability to construct a long-lived system that can be left unattended, (c) the rapidly decreasing cost of the wireless nodes and d) the ability to reconfigure their basic properties (e.g., routing tables, transmission power levels, *etc.*) make this technology be an ideal candidate for numerous applications [3–6]. WMSNs not only augment classic applications of WSNs but also facilitate new ones. Thus, the great variety of WMSNs applications includes surveillance and reconnaissance, environment and habitat monitoring, fire detection, inventory control, biological and biomedical applications, traffic control, energy management, monitoring and handling of emergency situations, and battlefield monitoring and control [1,7].

However, the current capabilities of wireless *ad hoc* sensor networks are not able to support the complete set of characteristics of wired multimedia networks. One basic problem has to do with the need for large bandwidths and high transmission data rates. More specifically, the requirement of such properties is in conflict with the basic idea behind the utilization of wireless sensor nodes (small bandwidth, reconfiguration, small power consumption) [8,9]. Actually, this is the primary reason which necessitates the development of Quality of Service (QoS) related algorithms, in order for a WMSN to be possible to continue operation, by avoiding the occurrence of bottlenecks that lead to network instability. As a result of this never-ending battle between bandwidth against miniaturization and power consumption, the current research work in the area of WMSNs has focused on problems such as: (a) resource constraints, (b) application-specific QoS requirements, (c) algorithms for high bandwidth demand, (d) variable channel capacity, (e) cross layer coupling of functionalities, (f) multimedia source coding techniques and (g) multimedia in network processing [7,10–13].

Many of the numerous applications of WMSNs are image-based. In such applications, sensor nodes have to capture, process and transmit huge amounts of optical data. However, the transmission of high volumes of data over a network increases the network traffic load and thus obstructs the prompt transmission of new image data and decelerates their refresh rate. In order to prevent these problems, the reduction of transmission rates could probably help. However, the prompt reception of image data is critical for most image-based applications of WMSNs. Thus, the reduction of transmission rates could make such applications be practically useless. An alternative solution is to reduce, via compression, the volume of the image data which are to be transmitted and maintain the transmission rates. However, this may considerably reduce the quality of the received images. Therefore, the image processing has to be efficient enough in order to make the best possible trade-off between the size of the images transmitted and their quality. Moreover, optical coding should have as little computational cost as possible for energy and time saving purposes.

The novel control scheme proposed in this article may be utilized in applications in which it is essential to retain the transmission of sequential image frames over a WMSN communication link, within soft bounded transmission delays, independently of the quality of the received images. The aforementioned demand of retaining the visual feedback, while penalizing the quality of the received image, is of paramount importance especially in semi-autonomous navigation functions in non-explored terrains based on communication links over wireless sensor networks. Moreover, the establishment of a control framework that provides soft-bounded time delays allows for the development of decentralized visual feedback serving applications in networked controlled systems, as the timing synchronization and the a priori bounds on the delays are of paramount importance [14–16].

More specifically, this article presents a novel congestion aware control scheme for the sequential transmission of image frames, through a WMSN. The implementation of the proposed congestion aware control scheme relies on: (a) the application of the Quad-Tree Decomposition (QTD) method [17], and (b) a gradient based adaptation of the quad tree decomposed factor, with respect to the identified congestion in the wireless sensor network, in order to retain the transmission delays within a priori defined time bounds. By inserting the QTD scheme, the acquired images, prior to their transmission, are QT-decomposed (compressed). This approach not only dramatically reduces the time for transmitting a single image frame, but also increases the flexibility level of the quality of the received images. More specifically, by utilizing the gradient-based adaptive scheme for increasing/decreasing the level of compression, the transmission times are correspondingly decreased/increased, while at the same time soft bounds on the latency times can be computed for further utilization in visual serving applications, while retaining the quality of the received images in as high levels as possible.

The remainder of this article is organized as follows. In Section 2, the QTD method is presented, while in Section 3, the proposed adaptive QoS mechanism for the image transmission is being introduced. The efficacy of the proposed control scheme is experimentally evaluated presented in Section 4, while in Section 5 the conclusions are drawn.

## 2. Quad Tree Decomposition Image Scheme

Typically, a natural image consists of some regions that locally have certain similarities and many other ones that have extensively varying content. Therefore, when coding such an image it is wise to allocate less data in order to decompose homogeneous neighborhoods and more data for areas containing edges and texture. In the scheme proposed in this work, a QTD scheme is utilized. QTD is an image segmentation method generally used for hierarchical decomposition. The main idea of hierarchical decomposition is to divide an image into sufficiently homogeneous areas, the levels of which can be compactly encoded. In the relevant bibliography, there are several image compression algorithms, with the most popular being the Discrete Cosine Transform, the Fractal compression, and the Wavelet Transform. The aforementioned techniques tend to be mathematically complex, except from the QTD algorithm. QTD has been widely utilized not only due to its low-complexity but because it is as well a powerful compression method [17–21].

These remarks make the QTD an ideal candidate for application in image based compression applications over WMSNs. In these applications most images are stored in raster format. Hence, any access to a raster image is sequential, starting from the top left-most pixel and ending at the bottom right-most pixel. The QTD can be performed in two alternative ways [21]. The first is the Bottom-Up decomposition where each image is initially segmented into blocks which have the minimum possible size. In sequel, every four adjacent blocks of equal size are joined together if the new joint block is homogeneous. The overall procedure is repeated until no other blocks can be merged. The second implementation approach is the Top-Down decomposition, where each image is initially divided into four blocks of equal size. Next, each of the newly generated blocks recursively splits into four new blocks if it is inhomogeneous and its size is greater than the minimum possible block size. In general, in terms of processing speed, the Top-Down QTD is considered to outperform Bottom-Up QTD for images which are either large or smooth while the latter performs better for images which have either small size or high texture.

**Figure 1.**
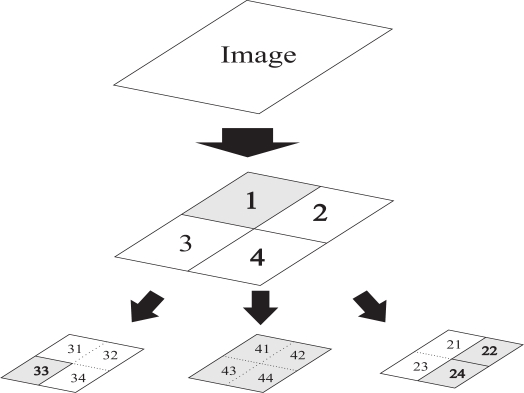
QTD example in the form of a multilayer set of blocks and a corresponding tree structure.

The image compression performed in this work, is based on the Top-Down QTD method. Thus, each image can be divided in half along both axes, all the way down to pixel level. This recursive subdividing of blocks allows the image data to be organized into groups, in accordance with the neighboring blocks. More specifically, every subdivision exists as one of four neighboring blocks. Actually, this is comparable to having a tree-like structure, where the root of the tree is the entire image, which recursively divaricates into four branches, until its leaves are pixels. In this manner, a quad tree is a tree having nodes which either are leaves (pixels) or have four children. As a result, each block is either completely a single color block or consists of four smaller sub-blocks. An example of a product of a QTD process is presented in Figure 1, where gray areas, are areas that no further decomposition can be applied. In the case presented in Figure 1, the original image frame has been decomposed into four blocks and sequentially the blocks 2, 3 and 4 have been further decomposed.

The tree-like evaluation of an image enables the removal of the unnecessary leaves and branches out of the tree, which result in the reduction of the QTD representation size. This could be achieved by checking every individual block whether it meets a criterion of homogeneity. If the former criterion is satisfied, the corresponding block is not divided any further. However, if the homogeneity criterion is not satisfied the block is further divided into another four blocks. The process is executed iteratively until each block satisfies the homogeneity criterion [22,23], expressed as:

(1) $$\text{max}({\mathrm{MX}}_{4}-{\mathrm{AVG}}_{4},\;{\mathrm{AVG}}_{4}-{\mathrm{MN}}_{4})\leq \frac{R}{L}(\frac{{\mathrm{AVG}}_{4}^{1-\frac{\gamma }{2}}}{q})$$

In this formula, *MX*_4_ represents the maximum value of the four leaves of a branch, while *MN*_4_ expresses the minimum value found on this branch, and *AV G*_4_ symbolizes the linear average of the values found on this specific branch. Additionally, *R* ∈ (0, 1) is the decomposition factor, which expresses the degree of compression, and *L* refers to a scaling factor which corresponds to the size of image region. For instance, *L*=1 corresponds to simple pixels, *L* = 2 corresponds to regions of size 2 × 2 and so on. Moreover, γ represents the gamma correction, and *q* denotes the ratio of the region to image size. For instance, when γ gets the commonly used value 2 and *q* is equal to 128, the quantity inside the parenthesis simplifies to 1/128. Thus, for a pixel array derived from an image of size 256 × 256, it represents 1/128 of the image size. This means that there are 128 pixel arrays in a 256 × 256 sized image. If a leaf is removed, a quadrant will be represented by the average of the pixels it contained before pruning. The utilization of the homogeneity criterion expressed by Equation (1), leads to the formation of images of reduced size created through the QTD.

**Figure 2.**
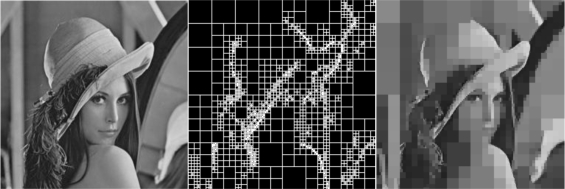
QTD process with *R* = 0.5 (Left: original image, Middle: QT-decomposed block partitioning, Right: Resulting QTD image).

The effect of decomposition factor on the quality of a QT-decomposed image is illustrated in Figure 2. More precisely, on the left side the of this figure an 8–bit gray scale image of benchmark image Lenna, which is commonly used in image processing research, with an analysis of 256 × 256 pixels is shown. In the middle portion of Figure 2 the block partitioning, resulting from a QT-decomposition with a decomposition factor *R* = 0.5 is presented. Finally, the right portion of the same figure contains the resulting QT-decomposed image of Lenna.

**Figure 3.**
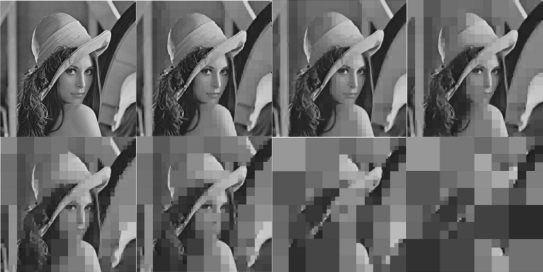
Progressive effect of the decomposition factor *R* on the image quality.

Based on the homogeneity criterion in Equation (1), it is straightforward that an increase/decrease of the decomposition factor *R* results in a corresponding decrease/increase of the quality/fidelity of QT-compressed image. To demonstrate this effect on the same Lenna benchmark image (utilized also in Figure 2), Figure 3 depicts the effect of the decomposition factor *R* on the QT-compressed image of Lenna. More specifically, the left most image on the upper row in Figure 3, is the original Lenna image. The images following this image from left to right at the upper row correspond to the cases where *R* takes sequentially the values 0.2, 0.3 and 0.4. Similarly, the images presented from left to right on the lower row of Figure 3 correspond to *R* values of 0.5 to 0.8.

The decomposition factor *R* affects not only the quality of the image but also its compression. The block partitioning of an image (blocks containing relevant pixels depending on the selected *R*) after a QTD is also affected by the selection of the *R* factor. Large/small number of blocks indicate a high/low image quality.

**Figure 4.**
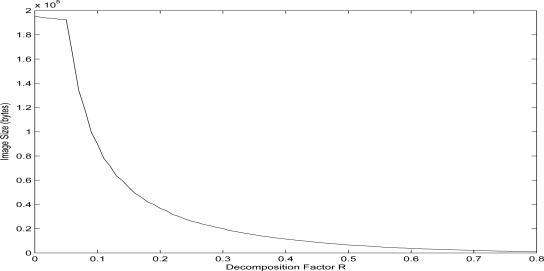
Effect of the decomposition factor *R* on the QTD resulting image size.

The decrease of the block partitioning directly decreases the data needed for the reconstruction of an image, and this is why by applying a varying decomposition approach in the transmitted images it is possible to control the corresponding time delays for transmitting a single image frame. This effect on the size of the resulting QTD image, is illustrated in Figure 4. More precisely, this figure depicts the relevance between the number of bytes needed for the representation of the image and the corresponding selected QTD factor *R*. The underlying assumption is that each block is characterized by four numbers. The first two numbers represent the position of the block in the image byte array, the third number indicates the size (square) of the block, while the fourth number indicates the value of the pixels contained in the block. For gray images, like the utilized benchmark image of Lenna, the pixels can take integer values in [0,255].

**Figure 5.**
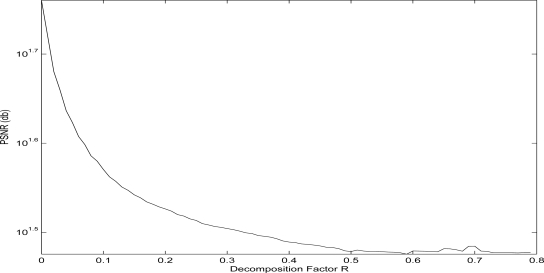
Effect of the decomposition factor *R* on the QTD resulting image quality.

The increase of the decomposition factor *R* correspondingly effects, except from the image size, the quality of the decomposed image. In order to measure this effect, the Peak Signal to Noise Ration (PSNR) measurement has been utilized. This PSNR ration expresses the difference in quality among the original Lenna image and the decomposed one, while the higher the PSNR is, the better the quality of the decomposed image is. For the examined case, the effect on the quality of the decomposed Lenna image *versus* the selected decomposition factor *R* is depicted in Figure 5.

## 3. Adaptive Image Compression for Transmission over a Wireless Multimedia Sensor Network

**Figure 6.**
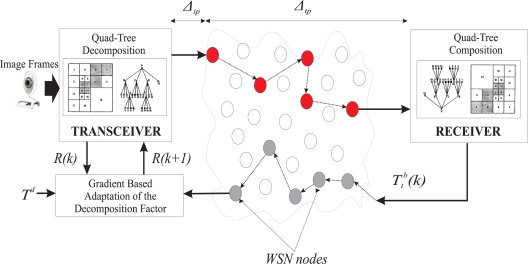
Architecture of the Image Transmission Scheme over a WMSN.

The proposed scheme, is an adaptive image compression algorithm, that regulates the quality of the transmitted images according to: (a) the traffic load within the network, and (b) the level of data contained in an image frame. The proposed image transmission scheme is based on the application of the QTD with a varying decomposition compression factor within a gradient adaptive approach, while the overall proposed architecture is depicted in Figure 6.

Initially, the images captured through a camera are QT-decomposed. Next, the resulting QTD partitioning of each single image is transformed in a batch *b* of data packets, in order to enable the image transmission over the employed WMSN. Through the multihop property of WSMNs the data packets reach the receiver side where the received data streams are composed into images.

The total transmission time needed, to complete the transmission of a single image frame, is considered as the summation of Δ*_ip_* time that represents a fixed inner-packet delay and the Δ*_tp_* time that denotes the packet’s transmission delay, where Δ*_ip_* ≪ Δ*_tp_*, and Δ*_ip_*, Δ*_tp_* ∈ ℜ^+^.

It is assumed that each data packet batch *b* has totally *S_b_* ∈ 𝒵^+^ bytes which are segmented in *N_b_* data packets having *p_b_j__* bytes per packet, where *p_b_i__* ∈ 𝒵^+^. The index *j* ∈ 𝒵^+^ denotes the varying size of bytes per packet. The total size of transmitted data, for a single image batch, can be calculated as:

(2) $$S_{b}=\sum_{j=1}^{N_{b}}p_{b_{j}}$$

In the case where the number of bytes per packet *p_b_i__* is constant and equal, i.e., all the *N_b_* data packets have the maximum full length *p_b_*, Equation (2) can be reformed as:

(3) $$S_{b}=N_{b}\cdot p_{b}$$

and the total transmission time $T_{t}^{b}\in {ℜ}^{+}$ needed for a complete data batch *b* to be transmitted, is given by:

(4) $$T_{t}^{b}=N_{b}\cdot (\Delta_{\mathrm{ip}}+\Delta_{\mathrm{tp}})$$

It should be noted that the value of Δ*_tp_* depends on the technical features of the hardware circuits utilized by the wireless sensors and thus it is considered to be constant for constant hardware setups. Δ*_ip_* represents the summation of all time delays caused by data transmission and can be analyzed in: (a) the traffic load within the network (congestion), and (b) the level of details contained in an image frame. From the previous analysis it is straightforward that the only variations, that affect the time needed for the complete transmission of an image, are due to the variations of Δ*_ip_*. This factor can be measured by applying time stamps in the data packets before transmission and requesting from the receiver for an acknowledgement signal.

Before the initialization of transmitting sequential image frames, an a priori bound *T^d^* is being set to the transmission time $T_{t}^{b}$ as:

(5) $$0\leq T_{t}^{b}\leq T^{d}$$

where *T^d^* ∈ 𝒵^+^ represents the maximum desired time delay that the transmission of a single image frame should not exceed. Let *e* ∈ ℜ be the deviation among the desired time delay *T^d^* and the currently measured time $T_{t}^{b}$, *i.e.*,

(6) $$e(k)=T^{d}(k)-T_{t}^{b}(k)$$

and the relative quadratic cost function:

(7) $$J={\left(T^{d}(k)-T_{t}^{b}(k)\right)}^{2}$$

where *k* ∈ 𝒵^+^ is the sample index. Let *μ* ∈ ℜ be the gradient convergence factor. Then, the following gradient based adaptation rule for the decomposition factor *R* can be derived:

(8) R(k+1)=R(k)−μ⋅e(k)

The overall algorithm is summarized in Table 1.

It should be noted that the proposed scheme addresses only the cases where there is a small variation of the captured image content, such as applications of surveillance and security. This limitation is mandatory for allowing the convergence of the proposed adaptation scheme. In the cases of fast content variations, even if a larger adaptation factor has been adopted, the proposed algorithm will not converge, the obtaining QTD-images will have a very poor quality, and the a priori soft time bounds will not be possible to be met. This limitation is not only a problem for the proposed adaptation scheme but it is a common problem in adaptive control and mainly in system identification that until now only partial solutions have been proposed for the cases of systems of slow varying dynamics [24].

In order to highlight the effects of the proposed adaptation scheme, the same image of Lenna has been utilized in all the experimental results depicted. This approach is providing more comprehensive and straight forward results to the reader, as the differences in the quality of the received QTD-images, *versus* the convergence factor, can be more easily identified, without loss of generality.

## 4. Experimental Results

The overall proposed QoS control mechanism has been applied in extensive experimental test cases. More analytically for the experimental test-bend, a video camera from Logitech, through a PC-104 Embedded module from AxiomTek has been connected to a MaxStream XBee XB24B Zigbee Modem, based on a XBEE USB connector board from Sparkfun. The video camera has been set in an 8-bit gray scale resolution, producing captured images images with an analysis of 256 × 256 pixels. This image has been also utilized to produce sequential transmitted images, in order to simplify the comprehension of the reader towards the variations in the quality of the sequentially received images, based on different decomposition factors *R*(*k*), without a loss of the generality for the presented approach.

The established Zigbee-WMSN consists of one coordinator node, three routers and one end device. The coordinator is responsible for maintaining the WMSN network and transmitting the decomposed images as data packets to the Zigbee network [25–27]. Moreover, the routers are responsible for establishing connections within the WMSN network in order to forward the decomposed image data packets (forwarders). The end device serves as the interface of the network to the computer at the receiver side.

The overall programming environment is NI’s LabView, while the XBee modems for the coordinator, the routers and the end device, as set up using the provided XBee API communication framework. The communication between the XBee modem and the computer are setup to a Baudrate of 9600 kbps using hardware flow control for the serial port. The selection of this data rate has been made again without loss of generality, as the presented experimental results can be also straight forward being extended to higher Baudrates. The parameters that have been utilized in the WMSN during the experiments performed are outlined in Table 2.

**Figure 7.**
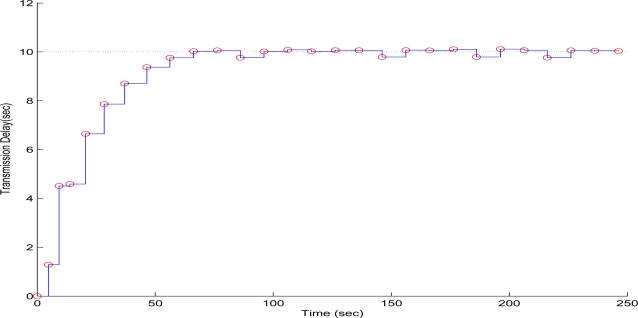
Measured Time Delay for transmitting an image frame over experimental time (Initial values *R* = 0.3, *T^d^* = 10 s, and *μ* = 0.01).

**Figure 8.**
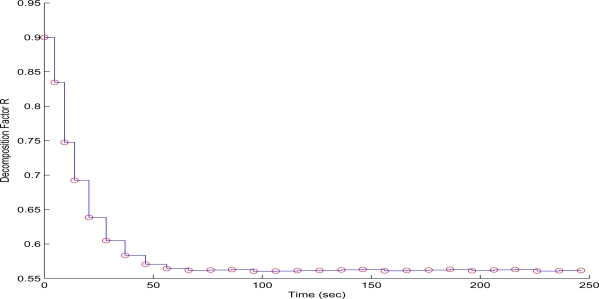
QT–Decomposition factor *R* convergence over experimental time (Initial values *R* = 0.3, *T^d^* =1 0 s, and *μ* = 0.01).

**Figure 9.**
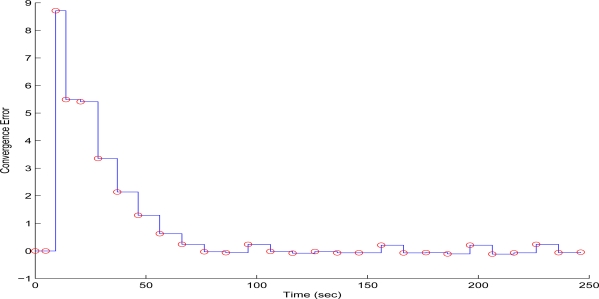
Convergence Error over experimental time (Initial values *R* = 0.9, *T^d^* = 10 s, and *μ* = 0.01).

To evaluate the performance of the proposed scheme in the first test case, sequential frames containing the 256 × 256 pixels gray scale and with a depth of 8-bit image of Lenna have been transmitted over the WMSN. The QT-decomposition factor is set to *R* = 0.9 (very high decomposition/very low quality of QTD image), the time bound for the overall transmission time was selected as *T_d_* = 10 s, while the initial value for the convergence factor *μ* was 0.01. In Figure 7 the time delay needed to transmit sequential image frames of Lenna with an adaptive *R* is presented, while in Figures 8 and 9 the corresponding time convergence of the QT-decomposition factor and the response of the error among the current measurement delay and the a priori bound are presented.

**Figure 10.**
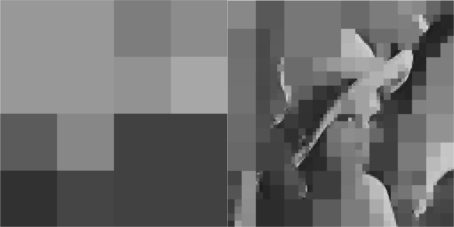
Initial Transmitted Image Frame (*t* = 0 s—left side)—Converged Transmitted Image Frame (*t* = 250 s—right side).

The selected initial value of *R* = 0.9 causes a high compression of the image and a very small transmission time (beginning of Figure 8) but with an extreme low quality in the transmitted image. Such a selection has been made in this article only for demonstrating the effectiveness of the proposed control scheme. Due to the gradient based, adaptation rule, the quality of the image is sequentially increased as *R* is decreased (Figure 8, while always retaining the soft bound that has been a priori set. As it can be observed in Figure 9 the proposed algorithm manages to converge in an *R* value that satisfies the boundary conditions for the transmission time delay, while maximizing the quality of the transmitted image. This can be also observed by Figure 10 where the initial transmitted image with *R* = 0.9 is compared against the final transmitted decomposed image with the converged value of *R* = 0.567, at the 250 s of the experimental results.

**Figure 11.**
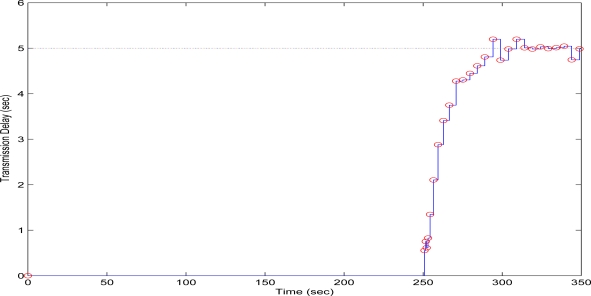
Measured Time Delay for transmitting an image frame over experimental time (Initial values *R* = 0.1, *T^d^* = 5 s, and *μ* = 0.01).

**Figure 12.**
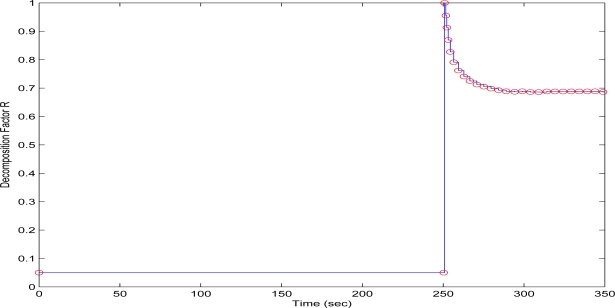
QT–Decomposition factor *R* convergence over experimental time (Initial values *R* = 0.1, *T^d^* = 5 s, and *μ* = 0.01).

**Figure 13.**
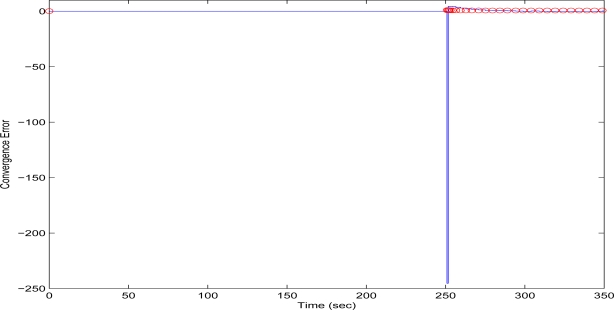
Convergence Error over experimental time (Initial values *R* = 0.1, *T^d^* = 5 s, and *μ* = 0.01).

In the second test case, the QT-decomposition factor was set as *R* = 0.1 (very low decomposition/very high quality of QTD image), the time bound for the overall transmission time was selected as *T_d_* = 5 s, while the initial value for the convergence factor *μ* was again set as 0.01. Again this selection of the decomposition factor has been made only for demonstrating the effectiveness of the proposed scheme, as such a selection achieves almost no compression in the transmitted decomposed image frame. In Figure 11 the time delay needed to transmit sequential image frames of Lenna with an adaptive *R* is presented, while in Figures 12 and 13 the corresponding time convergence of the QT–decomposition factor and the response of the error among the current measurement delay and the a priori bound are presented.

**Figure 14.**
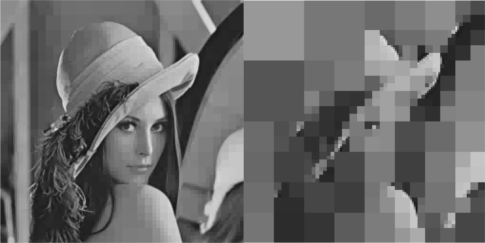
Initial Transmitted Image Frame (*t* = 0 s—left side)—Converged Transmitted Image Frame (*t* = 350 s—right side).

The initial value of *R* = 0.1 achieves low compression of the image and very large transmission time, where in this case it is noted that the second image frame, is being transmitted after 250 s, as it is also displayed in Figure 12. Due to the gradient based adaptation rule, the quality of the image is sequentially decreased as *R* is increased (Figure 12, while always retaining the soft bound that has been a priori set to 5 s. As it can be observed in Figure 13 the proposed algorithm manages to converge in an *R* value that satisfies the boundary conditions for the transmission time delay, while maximizing the allowed quality of the transmitted image. This can be also observed by Figure 14 where the initial image with *R* = 0.1 is compared against the final transmitted decomposed image with the converged value of *R*=0.6862, at the 350 s of the experimental results.

**Figure 15.**
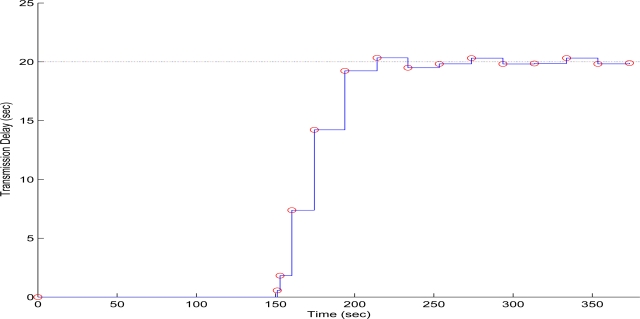
Measured Time Delay for transmitting an image frame over experimental time (Initial values *R* = 0.3, *T^d^* = 20 s, and *μ* = 0.01).

**Figure 16.**
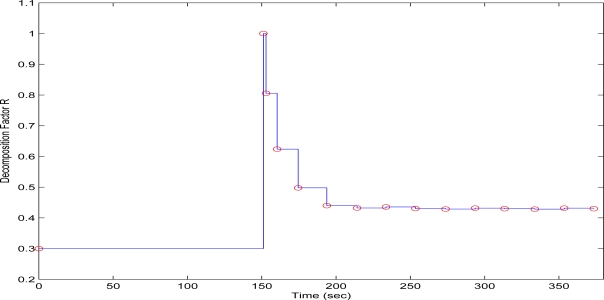
QT–Decomposition factor *R* convergence over experimental time (Initial values *R* = 0.9, *T^d^* = 10 s, and *μ* = 0.01).

**Figure 17.**
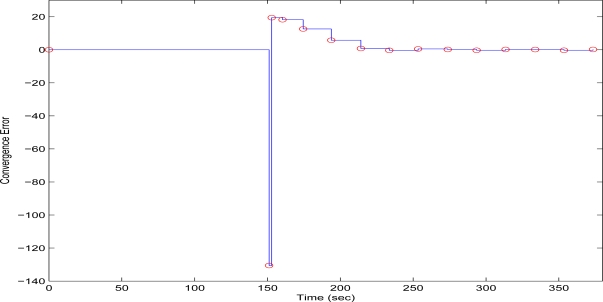
Convergence Error over experimental time (Initial values *R* = 0.9, *T^d^* = 10 s, and *μ* = 0.01).

Finally in the third experiment case, the QT-decomposition factor was set as *R* = 0.3 (medium decomposition/medium quality of QTD image), the time bound for the overall transmission time was selected as *T_d_* = 20 s, while the initial value for the convergence factor *μ* was again 0.01. In Figure 15 the time delay needed to transmit sequential image frames of Lenna with an adaptive *R* is presented, while in Figures 16 and 17 the corresponding time convergence of the QT-decomposition factor and the response of the error among the current measurement delay and the a priori bound are presented.

**Figure 18.**
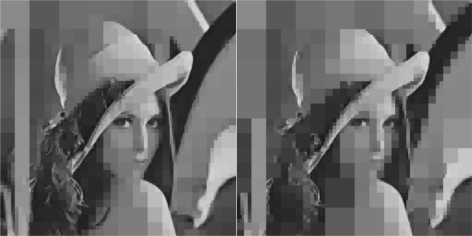
Initial Transmitted Image Frame (*t* = 0 s—left side)—Converged Transmitted Image Frame (*t* = 400 s—right side).

The initial selection of *R* = 0.3 is a factor that achieves a medium compression of the image while retaining acceptable transmission time delays. In Figure 16 is should noted that the selection of *R* = 0.3 results in a total delay of 150 s before transmitting the second image frame, a time duration that it is much longer than the a priori time bound. Due to the gradient based, adaptation rule, the quality of the image is sequentially decreased and the convergence of *R* is depicted in Figure 16, while the soft bound is always valid. As it can be observed in Figure 17 the proposed algorithm manages to converge in an *R* value that satisfies the boundary conditions for the transmission time delay, while maximizing the allowed quality of the transmitted image. This can be also observed by Figure 18 where the initial image with *R* = 0.3 is compared against the final transmitted decomposed image with the converged value of *R* = 0.430, at the 400 s of the experimental results.

**Figure 19.**
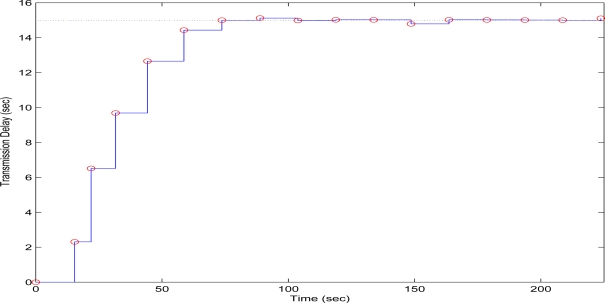
Measured Time Delay for transmitting an image frame over experimental time, under 10% congestion and packet losses (Initial values *R* = 0.8, *T^d^* = 15 s, and *μ* = 0.01).

**Figure 20.**
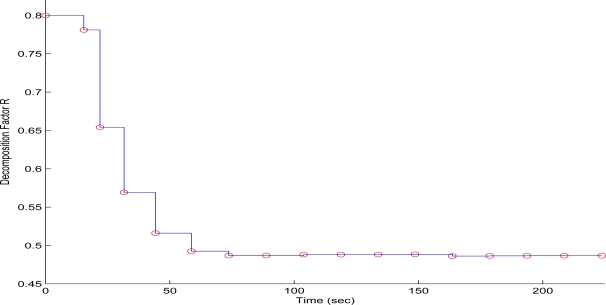
QT-Decomposition factor *R* convergence over experimental time, under 10% congestion and packet losses (Initial values *R* = 0.8, *T^d^* = 15 s, and *μ* = 0.01).

**Figure 21.**
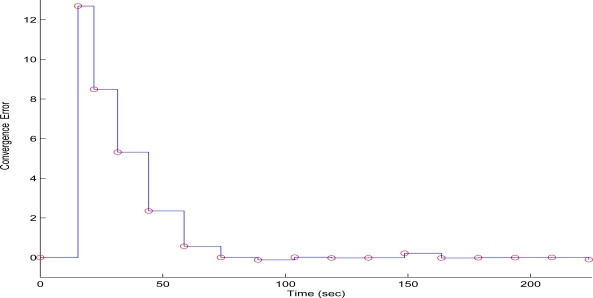
Convergence Error over experimental time, under 10% congestion and packet losses (Initial values *R* = 0.8, *T^d^* = 15 s, and *μ* = 0.01).

In what follows, the performance of the proposed scheme is demonstrated under the existence of congestion and packet losses. In this case, the distance among the nodes has been increased to 20 m and congestion has been considered in the transmission loop, as two other nodes transmit randomly data packets to the same receiver node. For the case of a 10% congested network, with an initial QT-decomposition factor of *R* = 0.8, an a priori time bound for the overall transmission time of *T_d_* = 15 *s* and for an initial values for the convergence factor of *μ* = 0.01, Figure 19 presents the time delay needed to transmit sequential image frames of Lenna *versus* the adaptation of *R*. Moreover Figures 20 and 21 present the corresponding time convergence of the QT-decomposition factor and the response of the error among the current measurement delay and the a priori bound respectively.

**Figure 22.**
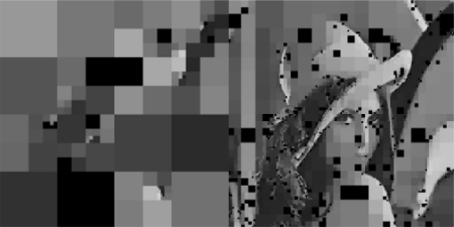
Initial Transmitted Image Frame (*t* = 0 s—left side)—Converged Transmitted Image Frame (*t* = 225 s—right side) under 10% Network Congestion.

As it can be observed in Figure 22, where the initial image with *R* = 0.8 is compared to the final transmitted decomposed image, with a converged value of *R* = 0.4866, the existing congestion and packet losses do not influence the quality of the overall proposed scheme and more specifically the quality of the received image. This fact has been the main reason for selecting the QT-Decomposition, as losses during the data packet transmissions, result only in the loss of small image details and not in a complete loss of the data included in the transmitted image. This is more obvious in Figure 22 where it can be observed that the data packet losses have appeared in the received image as square black areas.

**Figure 23.**
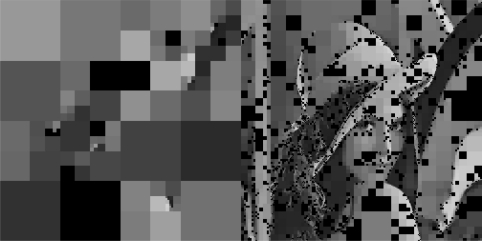
Initial Transmitted Image Frame (*t* = 0 s—left side)—Converged Transmitted Image Frame (*t* = 227 s—right side) under 20% Network Congestion.

As it can be observed in Figure 23, same results can also be obtained for the case of a 30% congested network, and with the same characteristics and initial conditions as before. Despite the increase of the data packet losses, the quality of the image still remains satisfactory.

As it has been shown through the experimental results, the presented adaptation scheme has the advantage of being completely scalable. This means that addition of multiple image senders and multiple image receivers respectively would not alter the functionality of the proposed scheme, except from the levels of congestion and packet losses. In the case of multiple image sender nodes and one image receiver node the proposed scheme will result in: (a) a significant increase of congestion and packet losses, (b) increase of the round trip times for a complete transmission of an image frame, (c) increase of the overhead in the data packets due to the need of tagging the data packets with respect to the image that belong, and this is why such an approach is not recommended.

From the experiments and the relevant analysis presented it is derived that the proposed control scheme is ideal for transmitting sequential images with provided soft bounds on the transmission time delays, specifically in the cases where the sequential frames are containing images without extreme changes in their data content.

## 5. Conclusions

In this article a sequential image transmission scheme over Wireless Sensor Networks (WSN) with soft bounded transmission time delays has been presented. The proposed control architecture, was a Quality of Service (QoS) algorithm, that adapts the quality of the transmitted images, in a gradient based adaptive approach, in order to provide soft bounds for the image transmission time delays (end to end). The bounding of the transmission time, needed for a single frame, has been achieved by applying Quad Tree Decomposition (QTD) with a varying decomposition compression factor. For the initialization of the proposed control scheme, the desired a priori maximum bound for the transmission time delay is being set, while a trade-off among the quality of the decomposed image frame and the time needed for completing the transmission of the frame should be taken under consideration. The efficacy of the proposed QoS control scheme was evaluated through extended experimental results.

## Figures and Tables

**Table 1.** Gradient Based Adaptive QoS Scheme Algorithm.

1. Define the desired *T^d^*
2. Select a convergence factor *μ*
3. Select the initial value for the decomposition factor *R*(*k*)
4. Decompose an image frame and construct the decomposition matrix that contains the data packets
5. Construct the *i^th^* data packet batch *S_b_*
6. Start the transmission of the first batch
7. Measure the time $T_{t}^{b}$ needed to transmit the *i^th^* data bath
8. Calculate the current error: $e\left(k\right)=T^{d}\left(k\right)-T_{t}^{b}\left(k\right)$
9. Perform the gradient based adaptation rule: *R*(*k* + 1) = *R*(*k*) − *μ*·e(k)
10. Return to step 4

**Table 2.** Experimental configuration of the utilized WN

**Network Characteristics**	**Values**
Number of nodes (*N*)	6
Coverage Area (*m* × *m*)	15 × 15
Maximum Transmission Range (m)	40
MAC Sub-Layer Protocol	IEEE 802.15.4
Routing Protocol	ZigBee
Data size per Packet (B)	68
Data size per Packet (including Overhead) (B)	84
Distance between nodes(m)	4
Interface Baudrate (Kbps)	9600
Interface flow control	Hardware (CTS/RTS)
RF Data rate (Kbps)	250
Transmit output power (mW)	1.25
